# Pulp analysis of teeth submitted to different types of forces: a histological study in rats

**DOI:** 10.1590/1678-7757-2017-0626

**Published:** 2018-10-02

**Authors:** Osmar Aparecido Cuoghi, Lorraine Perciliano de Faria, Edilson Ervolino, Sônia Regina Panzarini Barioni, Francielle Topolski, Victor Elias Arana-Chavez, Marcos Rogério de Mendonça

**Affiliations:** 1 Univ. Estadual Paulista, Faculdade de Odontologia de Araçatuba, Departamento de Odontologia Infantil e Social, Araçatuba, São Paulo, Brasil.; 2 Universidade de São Paulo, Faculdade de Odontologia, Departamento de Biomateriais e Biologia Oral, São Paulo, São Paulo, Brasil.; 3 Univ. Estadual Paulista, Faculdade de Odontologia de Araçatuba, Departamento de Ciências Básicas, Araçatuba, São Paulo, Brasil.; 4 Univ. Estadual Paulista, Faculdade de Odontologia de Araçatuba, Departamento de Cirurgia e Clínica Integrada, Araçatuba, São Paulo, Brasil.

**Keywords:** Tooth movement, Dental pulp, Histology

## Abstract

**Objective:**

The purpose of this study was to histologically evaluate pulp and dentin under induced tooth movement (ITM) with different types of forces.

**Material and Methods:**

The maxillary right first molars of rats were submitted to movement with continuous (CF), continuous interrupted (CIF) and intermittent (IF) forces during 5, 7 and 9 days with nickel-titanium (NiTi) closed-coil springs exerting 50cN force magnitude. The groups were histologically evaluated as for cellularity pattern, presence of dystrophic, hemodynamic alterations in the pulp as well dentin alterations. The main observed alterations were related to hemodynamic pulp characteristics, such as presence of thrombosis, vascular congestion and hemorrhages. The hemodynamic alterations were statistically evaluated by Shapiro-Wilk normality test and analysis of variance by the Kruskall-Wallis test.

**Results:**

There was no significant differences observed between groups in the different types of applied forces and duration of ITM (vascular congestion, p=1.000; hemorrhage, p=0.305; thrombosis, p=1.000).

**Conclusions:**

Pulp tissue alterations resulting from ITM were limited to hemodynamic events, without progressing to irreversible degeneration, regardless of the type of force applied.

## Introduction

Periodontal and dental pulp alterations may be induced by orthodontic movement, according to magnitude, frequency and duration of the applied force. ^1^ In orthodontics, the types of applied forces are continuous (CF), continuous interrupted (CIF) and intermittent (IF). ^2^ Studies on tissue alterations resulting from induced tooth movement (ITM), with different types of applied forces, have mainly focused on periodontal reactions and only a few have addressed pulp alterations. ^3^ ^-^ ^12^

The dental pulp is a highly vascularized loose connective tissue, presenting a great number of cells, extracellular matrix, blood vessels and nerve fibers. Similar to other connective tissues, the pulp has an elevated repair capacity, recovering easily under favorable conditions. The dental pulp and dentin are derived from the ectomesenchyme of the dental papilla. The coronal pulp is protected from external agents by the dentin and more externally by the enamel. On the other hand, this protection is converted into threat compromising its own existence. Thus, in face of any physical, chemical or bacterial aggressor agent, whose stimuli exceed the threshold of physiologic tolerance, an inflammatory and/or degenerative pulp response may occur. These alterations result in significant intrapulp pressure increase, due to histological characteristics of the dental pulp. ^13^

ITM is associated with some alterations of the dentin-pulp complex, such as rupture of the odontoblastic layer, alterations in microcirculation, hypoxia and pulp calcifications. ^9^ ^,^ ^14^ Depending on the type, magnitude and duration of the applied force, as well as the physiologic degree of tolerance of the dental pulp, it can be reversibly or irreversibly altered. ^10^ The extension of the dental pulp lesion is also correlated with the degree of the produced inflammatory response that is mediated by neuropeptides. ^15^

Studies have associated extrusive orthodontic forces with some harmful effects on the pulp and periodontal tissues, such as vascular stasis and pulp necrosis. ^16^ On the other hand, intrusive forces have been associated with vascular alterations, increase in fibrosis and calcifications. ^17^ In order to prevent the harmful effects on the tooth pulp, the recommended applied force magnitude in adults has been between 50 and 100 cN. ^18^

While some studies showed no pulp alterations resulting from orthodontic tooth movement, ^19^ ^,^ ^20^ other studies found several events, such as invasion of macrophages, cellular proliferation, vascular alterations and disorganization of the odontoblast layer. ^3^ ^-^ ^12^ A few cases of tooth vitality loss during the orthodontic treatment were reported; however, when present, they were associated with previous traumas and/or poor control of the orthodontic force. ^21^

Recent systematic reviews demonstrated that scientific evidences do not support the correlations between type of applied orthodontic force and dental pulp reactions. ^11^ Besides, only intrusive, extrusive and tipping forces were previously considered, without comparing CF, CIF and IF. ^11^ ^,^ ^22^

Considering the principle that biological stimuli promotes tooth movement, ^23^ it is important to carry out a study that examine the tissue alterations resulting from ITM, in order to better delineate the orthodontic treatment. Therefore, the purpose of this study was to histologically evaluate the dental pulp after ITM using different types of forces and treatment periods by using a suitable rat model.

## Material and methods

### Animals

The experimental procedures were approved by the Institutional Animal Ethics Committee of the School of Dentistry of Araçatuba (Sao Paulo State University - UNESP, SP, Brazil), process number 01247-2015.

Fifty-four young adult male Wistar rats *(Rattus norvergicus, albinus)* obtained from the animal facility (School of Dentistry of Araçatuba, - UNESP) were used in the present study. The animals were kept in cages with six animals each, with free access to food and water, under 12/12 hour light cycle and temperature of 22°C±2°C.

### Experimental design

The experimental procedure was divided into two phases. First of all, the rats at 62 days old were submitted to ankylosis induction of maxillary right incisor in order to obtain better control of the molar movement.

This phase was performed according to a predetermined protocol ^7^ following the steps: anesthesia with ketamine chlorohydrate (80 mg/kg - Dopalen, Sespo Ind. & Com. Ltda., Jacareí, SP, Brazil) and xylazine hydrochloride (10 mg/kg - Anasedan, Agribrands do Brazil Ltda., Paulínia, SP, Brazil), followed by luxation and extraction of the maxillary right incisor. After that, periradicular tissues (periodontal ligament) and dental papilla were removed and the root canal was obturated with a mixture of calcium hydroxide and propylene glycol. Then, the root apex was sealed with MTA (Angelus, Londrina, PR, Brazil) and reimplantated. A period of two weeks was allowed for formation of ankylosis before the second phase was initiated.

The second phase consisted of ITM of the maxillary right first molar when the rats were 76 days old, with a mean weight of 300 g. The animals were divided into three groups according to the types of applied forces: continuous (CF), continuous interrupted (CIF) and intermittent (IF). The teeth were moved for 5, 7 and 9 days. The groups were subdivided and identified as CF5, CF7, CF9, CIF5, CIF7, CIF9, IF5, IF7, IF9, with six animals *per* group.

**Figure 1:**
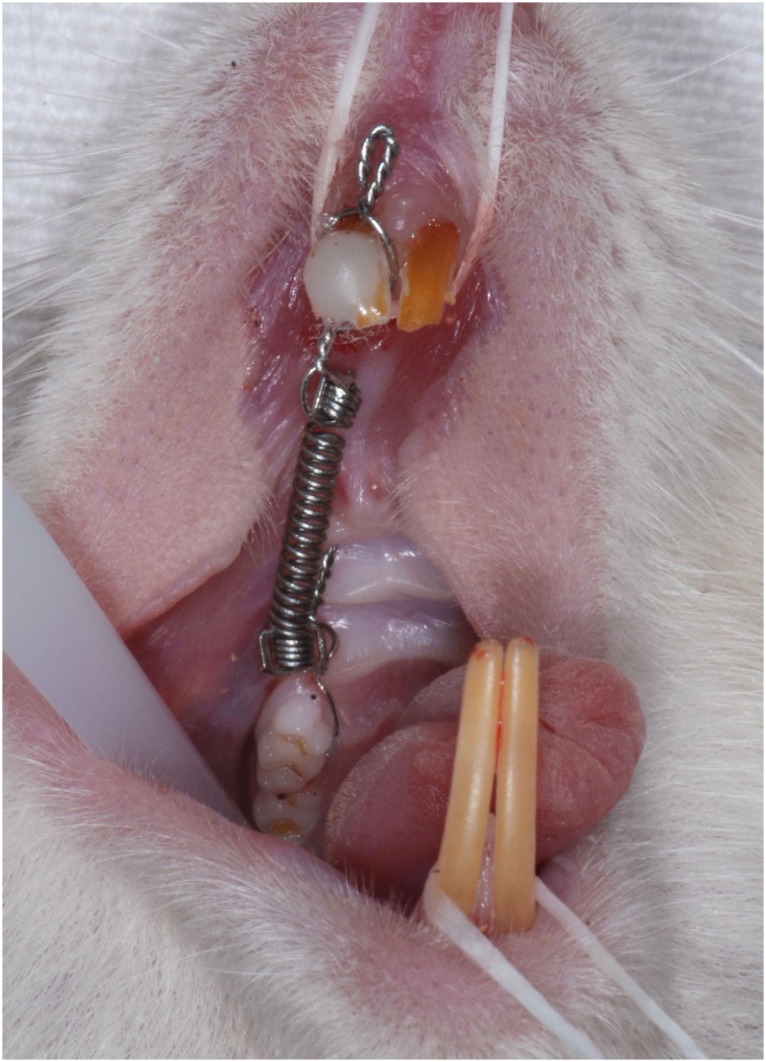
Intraoral photograph of nickel-titanium closed-coil spring between the right maxillary first molar and maxillary incisors of the rat

For movement of the first molars, the incisors were used as anchorage, similar to the device idealized by Heller and Nanda ^24^ (1979). A nickel-titanium closed-coil spring (Sentalloy, GAC, NY, USA) exerting 50 cN was used ^25^ ( Figure 1 ).

**Figure 2:**
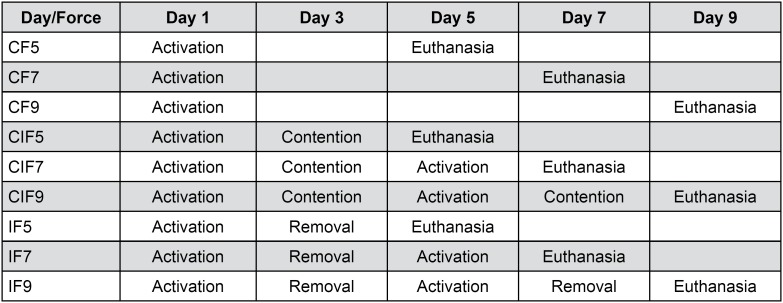
Distribution of experimental groups

In order to obtain continuous force (CF), the spring was activated excerting 50 cN during the whole movement period. In CIF group, continuous interrupted force was obtained through periods of activation (spring was activated excerting 50 cN) and inactivation (the spring remained passive, without excerting force). To intermittent force (IF), the spring went through periods of activation and removal ^25^ ( Figure 2 ).

### Histological analysis

Animals were anesthetized by overdose and sacrificed by decapitation 5, 7 and 9 days after starting the force application. The maxillary blocks, from the incisors to the third molars, were set in 10% formalin for 48 h, washed in running water for 24 h and decalcified in an 18% E.D.T.A. solution for 6 weeks, dehydrated, clarified, and embedded in paraffin blocks. Histological cross sections of 6 μm showing the radicular cervical level of the molars (exhibiting the separation of all roots) were stained with hematoxylin and eosin (HE).

**Figure 3:**
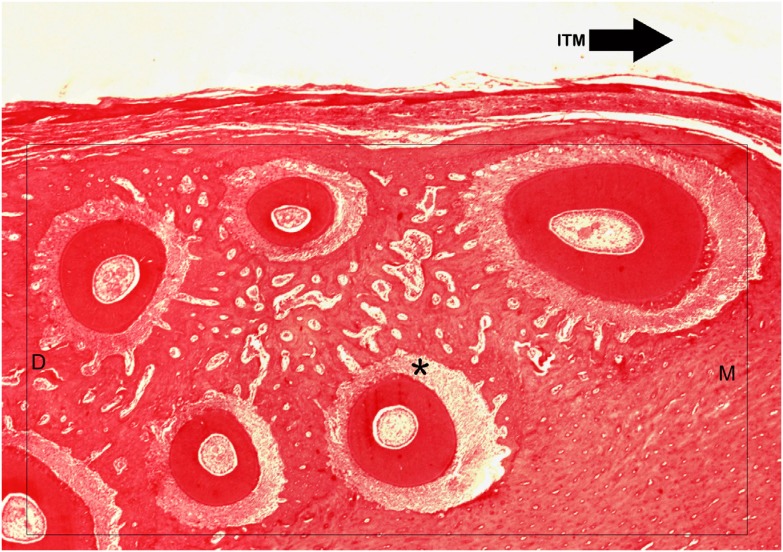
Light micrograph showing the roots of the right maxillary first molar tooth which was submitted to ITM. In black arrow the direction of ITM, in (*) the mesiopalatal root, in (M) mesial and in (D) distal side. Hematoxylin and eosin magnified 2.5x

Histological images were captured using an optical light microscope (Carl Zeiss, Göttingen, Germany), coupled to a digital camera (Axio Cam MRc5, Carl Zeiss, Göttingen, Germany). The radicular pulp of five roots of the right first molar ( Figure 3 ) was evaluated following an adaptation of the method proposed by Massaro, et al. ^19^ (2009). The following histological characteristics were evaluated: presence or absence of inflammatory infiltrate, reduced cellularity, increased fibrosis, pulp hyalinization, pulp nodules, diffuse calcification, necrosis, vascular congestion, hemorrhage, thrombosis, reactional dentin, tubules with nuclei.

### Statistical analysis

Thirty-days after the first histological evaluation, 10% of the sample was drawn (n=6) using Excel spreadsheets and repeating the evaluations. The obtained results of the first and second evaluations were submitted to Kappa test, for intra-examiner calibration analysis. The Kappa coefficient index was 0.637, demonstrating a good agreement level. The data were analyzed using the Sigma Plot software program (Systat Software Inc., San Jose, CA, USA) and submitted to the normality test (Shapiro-Wilk) and to analysis of variance by the Kruskall-Wallis non-parametric test at 5% significance level (p<0.05).

## Results

**Figure 4:**
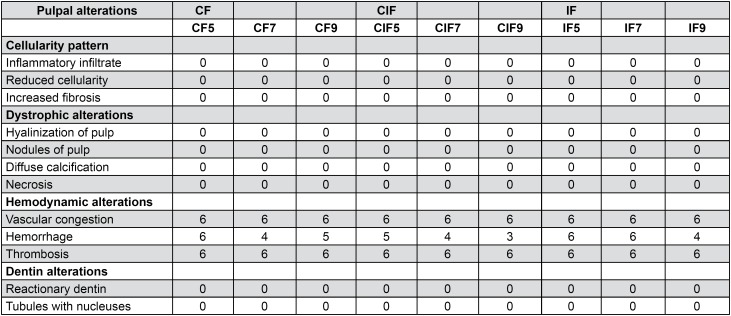
Frequency of microscopically observed pulp phenomena in the groups submitted to ITM with CF, CIF and IF

The histological findings were divided into cellularity pattern (inflammatory infiltrate, reduced cellularity, increased fibrosis), presence of dystrophic alterations (pulp hyalinization, pulp nodules, diffuse calcification, necrosis), hemodynamic alterations (vascular congestion, hemorrhage, thrombosis) and dentin alterations (reactional dentin, tubules with nucleuses) ( Figure 4 ).

*All groups: n=6

**Figure 5:**
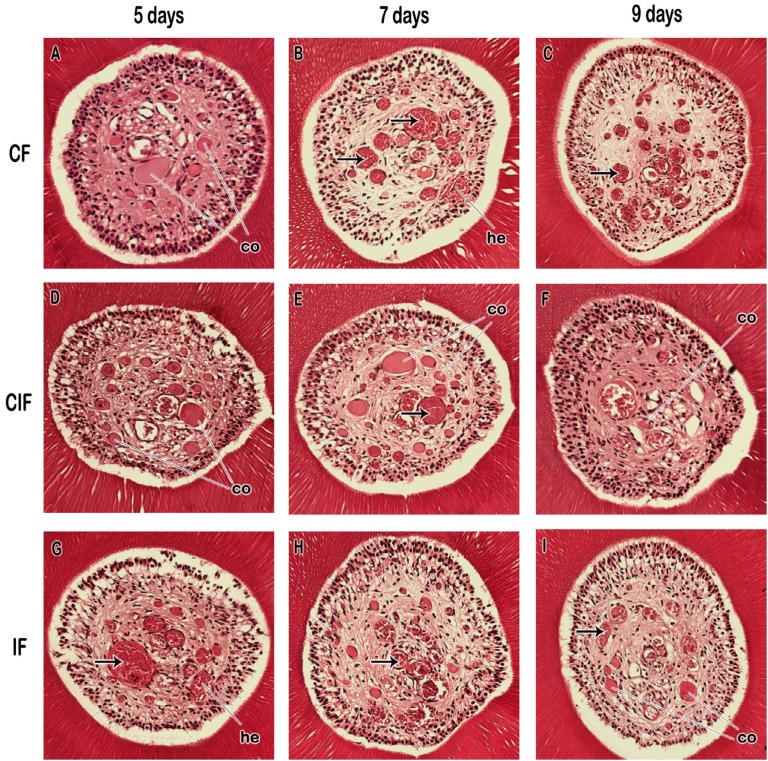
Light micrographs showing the mesiopalatal root of the right maxillary first molar tooth which was submitted to ITM in 5, 7 and 9 days with continuous force (CF), continuous interrupted force (CIF) and intermittent force (IF). Evidence of thrombosis in black arrow, vascular congestion (co) and hemorrhage (he). Hematoxylin and eosin magnified 20x

The cellularity and extracellular matrix organization pattern remained unchanged, with absence of inflammatory infiltrate in all groups and periods. Inflammatory cells were within the normality pattern ( Figure 5 ).

Dystrophic alterations such as presence of hyalinization, necrosis, diffuse calcification and pulp nodules were not observed in any of the groups and studied periods ( Figure 5 ).

Vascular congestion and thrombosis were similar in all the studied animals. Hemorrhage was more frequent at day 5 of ITM in all groups and with lower frequency in the group submitted to CIF at day 9 ( Figure 5 ). No significant differences (vascular congestion, p=1.000; hemorrhage, p=0.305; thrombosis, p = 1.000) were observed between groups in the different types of applied forces and duration of ITM.

Dentin alterations such as presence of tubules with cellular nuclei and formation of reactional dentin were not observed in any groups and studied periods ( Figure 5 ).

## Discussion

The pulp lesion observed during ITM was classified as mild and transitory, since it does not cause necrosis or abscess, but it is able to cause inflammatory reaction. ^26^ This process seems similar to that found in humans during orthodontic movement when intense forces are used. ^5^ Vascular alterations in the pulp were associated with ITM ^5^ ^-^ ^7^ and can be triggered by neuropeptides, defined as neurotransmitters or neuromodulators. ^27^ Some neuropeptides can induce vasodilation, plasma leakage, immune system activation, chemotaxis and recruitment and/or regulation of inflammatory cells, such as macrophages, mastocytes and lymphocytes. ^4^

Neuropeptides such as the calcitonin gene-related peptide (CGRP) can be triggered by caries, traumas, and also by the action of orthodontic forces. CGRP increases the bone morphogenetic protein (BM P) expression in human pulp cells, stimulating dentin deposition by odontoblasts as a defense mechanism. This event, along with hypoxia, induces degenerative calcification of the dental pulp and might cause obliteration of radicular pulp. ^3^ ^,^ ^8^ ^,^ ^9^ However, dentin alterations such as reactional dentin deposition, dentin organization, as well as presence of dental pulp calcifications were not noticed in the present study.

Mild forces can cause a small release of CGRP, leading to an initial vascular congestion. In this study, the hemodynamic event was frequently observed in all groups; however, significant differences did not occur between the different types of forces. Nevertheless, vascular congestion can be compensated by release of angiogenic factors, preventing irreversible damages from occurring to the pulp. ^15^ ^,^ ^28^ ^,^ ^29^

Hemodynamic alterations such as presence of congested, thrombotic vessels and some hemorrhage areas showed an increased tendency in this study. These events can occur due to pulp microcirculation changes, which increase tissue pressure, leading to rupture of the vessel epithelium, causing hemorrhages. ^10^ The hemodynamic alterations may be triggered by inflammation (irreversible pulpitis) and culminate in pulp necrosis. Coagulation necrosis may also occur due to thrombi or vessel rupture, in case of absence of blood supply. ^13^ Santamaría, et al. ^24^ (2007), evaluating pulp alterations at 6, 12, 24 and 72 hours after ITM, also did not observe any case of necrosis, similar to the condition observed in the present study. The effects of ITM on the pulp, already begin a few hours after the beginning of force application. ^5^ ^,^ ^7^ However, some alterations take a longer time to be detected, such as the deposition of reactionary dentin. Therefore, we chose to study the periods of 5, 7 and 9 days, aiming to observe not only cellular changes, but also structural alterations. Aguiar and Arana-Chavez ^30^ (2007), found that after 7 days of minor trauma (extrusion), is already possible to see the start of the formation of a tubular dentine matrix (reactionary dentine). With 10 days, they observed areas of tubular reactionary dentine were coated with original odontoblasts layer. Younger dental pulps are larger, exhibiting a great number of cells with little or no fibrosis. This histological pattern changes with time and the dental pulp reduces its volume due to deposition of secondary, reparative or reactional dentin, increasing fibrosis and cellular density, besides reducing blood vessels. ^13^ These dynamic alterations may explain the slight pulp alteration observed in this study, given that the animals were young and the dental pulp presented a greater capacity of reaction to environmental variations, like the ITM force stress. Furthermore, nodules and pulp calcifications are part of the natural “aging” process of the pulp, however, they can occur earlier in face of traumatic processes on the teeth structure. ^31^ These events were not observed in the present study, suggesting that the age of animals, intensity, type and duration of the applied forces were biologically acceptable and produced dystrophic alterations. Although the force used to promote tooth mesialization may yield tipping or bodily movement. The device used in this study promotes tipping forces, which depending on the intensity of the stimulus, can reduce blood flow, causing various damages. ^32^ Furthermore, studies comparing tipping and bodily forces with 10, 25, 50 e 100 g, had shown that tipping forces may cause more deleterious effects than bodily. ^32^ ^,^ ^33^

Orthodontic treatments with continuous force, without interruption, are frequently adopted in daily practice. Even at low force magnitude, the harmful effects may occur at higher frequency, due to either anatomical bone, dental and individual periodontal variety, besides stress continuously acting for a long period in the periodontal ligament. Cuoghi, et al. ^34^ (2014) evaluated the effects of CF, CIF and IF in adult rats by histological analysis. These authors found higher root resorptions associated with CF, corroborating data that duration plays an important role in the biological mechanism, promoting different tissue reactions. ^1^ Massaro, et al. ^19^ (2009) observed higher radicular resorption at day 7 of ITM with continuous force; however, without greater pulp damage. Further studies are needed to investigate whether radicular resorption can be associated with pulp alterations in later periods after tooth movement.

Under the conditions of the present study, it may be concluded that dental pulp alterations after ITM were limited to hemodynamic events, without significant differences between the studied groups, regardless of type and duration of applied force. No signs of progression to irreversible dental pulp degeneration were observed.
